# Basal cell adenoma with S100 protein–positive “stroma”: a distinct triphasic salivary gland neoplasm characterized by *CTNNB1* mutation

**DOI:** 10.1007/s00428-025-04141-2

**Published:** 2025-06-14

**Authors:** Alena Skálová, Martina Bradová, Jan Laco, Tomáš Vaněček, Veronika Hájková, Petr Martínek, Marián Grendár, Giulia Querzoli, Ilmo Leivo, Michal Michal

**Affiliations:** 1 Department of Pathology, Faculty of Medicine, Sikl’s, Charles University, E. Benese 13, 305 99 Plzen, Czech Republic; 2 Bioptic Laboratory, Ltd, Plzen, Czech Republic; 3 The Fingerland Department of Pathology, Faculty of Medicine, Charles University, Hradec Kralove and University Hospital Hradec Kralove, Hradec Kralove, Czech Republic; 4 Molecular and Genetic Laboratory, Bioptic Laboratory, Ltd, Plzen, Czech Republic; 5 Pathology Unit, IRCCS Azienda Ospedaliero Universitaria Di Bologna, Bologna, Italy; 6 Department of Medical and Surgical Sciences (DIMEC), University of Bologna, Bologna, Italy; 7 Institute of Biomedicine, Pathology, Department of Pathology, University of Turkuand, Turku University Hospital, Turku, Finland

**Keywords:** Salivary gland, Basal cell adenoma, S100 protein–positive stroma, Molecular diagnostics, Next-generation sequencing, Methylation analysis

## Abstract

Basal cell adenoma (BCA) is a benign salivary neoplasm that exhibits a divergent spectrum of growth patterns, including cribriform, tubular, trabecular, membranous, and solid. A subset of BCAs is characterized by the presence of abundant S100 protein–positive stroma, which makes this variant unique and potentially represents a hybrid lesion or an entity intermediate between BCA and pleomorphic adenoma (PA). From the authors’ registry, we selected 17 cases of BCA with abundant S100 protein–positive stromal components and compared them with 7 cases of BCA without S100 protein–positive stroma, and 6 cases of myoepithelial cell–rich PAs. All cases were analyzed by immunohistochemistry (IHC) using antibodies to S100 protein, SOX10, PLAG1, HMGA2, p63/p40, cytokeratins, EMA, LEF1, and/or β-catenin. Next-generation sequencing (NGS), fluorescence in situ hybridization (FISH) for the rearrangement of *PLAG1*, and methylation analysis were performed. The BCA S100 protein stromal cell–rich group consisted of 7 males and 10 females with an average age of 62 years. Their tumors showed typical S100 protein–positive stroma, which was also positive for SOX10 in all cases. The stromal and/or epithelial components showed expression of LEF1 and β-catenin in 17 and 15 cases, respectively. HMGA2 IHC showed nuclear expression in one case while PLAG1 was negative in all cases. In 11 cases, one or more mutations were present, including *CTNNB1* mutation (*n* = 11). The first control cohort of BCA without S100 protein–positive stroma consisted of 1 male and 6 females with an average age of 50 years. This group showed LEF1 and nuclear β-catenin expression in 1 and 2 cases, respectively. The second control group of PA (including 4 spindle-shaped cellular and 2 oncocytic PAs) was devoid of *CTNNB1* mutations. Two cases presented with gene fusions, including *MEG3::PLAG1* and *ACTA2::PLAG1*, and an additional two cases showed *PLAG1* break. It has been proposed earlier that BCA is related to PA based on a shared biphasic nature and a divergent spectrum of growth patterns. Our findings suggest that BCAs with abundant S100 protein–positive stroma are tumors that morphologically display tricellular differentiation into inner (luminal) ductal epithelial cells, outer (abluminal) basaloid myoepithelial cells, and spindle-shaped stromal S100-positive cells (stromal abluminal). According to our investigation, BCAs with S100 protein–positive stroma represent a distinctive triphasic subset of BCA, which is substantially different from PA, both in immunoprofile and molecular underpinnings.

## Introduction

Basal cell adenoma (BCA) is a rare benign biphasic salivary neoplasm that exhibits a divergent spectrum of growth patterns, including cribriform, tubular, trabecular, membranous, and solid. BCA is also characterized by a basaloid appearance due to the high nuclear-to-cytoplasmic ratio of neoplastic cells, a jigsaw puzzle growth pattern, and peripheral cell palisading [1]. It was proposed earlier that BCA is related to pleomorphic adenoma (PA) based on shared biphasic nature and a divergent spectrum of growth patterns [2]. By definition, the key histologic feature separating BCA and PA is the absence of a myxochondroid stroma in the former [1]. A subset of myoepithelial cell–rich PA devoid of myxoid and chondromyxoid stromal component mimicking BCA is, however, a well-known pitfall [3, 4], opening the question of a possible relationship between BCA and PA.

BCA with abundant myoepithelial cell–derived S100 protein–positive “stroma” was described by Dardick et al. in 1986 for the first time as a distinct salivary tumor entity [2]. Dardick et al. proposed that such a subtype of BCA might represent a variety of cellular PA or, at least, a type of hybrid lesion between BCA and PA [2]. For many years, this proposal was forgotten and not discussed in the literature. BCA can morphologically overlap with all basaloid biphasic salivary tumors, but once its benign nature is established, the main distinction is between BCA and cellular PA. In particular, BCA with a prominent S100 protein–positive stromal cells can mimic a cellular PA. However, unlike in cellular PA, the stroma in a BCA does not blend into the epithelial nests but remains distinct instead. Nuclear β-catenin immunoexpression may also help to distinguish these two entities [5].

In recent years, considerable progress in salivary gland tumor taxonomy has taken place with the discovery of tumor type-specific fusion oncogenes generated by chromosomal translocations and tumor type-characteristic gene mutations [6, 7]. BCAs frequently harbor *CTNNB1* mutations [5] while most cases of PA are characterized by alterations in *PLAG1* [8] or *HMGA2* genes [9, 10]. Moreover in recent years, DNA methylation has emerged as a further approach for tumor classification and has been initially established for brain [11] and soft tissue tumors [12].

A subset of BCA is characterized by the presence of abundant S100 protein–positive abluminal spindle-shaped stromal cells, which makes this variant unique and potentially represents a hybrid lesion or an entity intermediate between BCA and PA/myoepithelioma. Recently, Jurmeister et al. proposed a DNA methylation–based classifier of salivary gland tumors [13], and demonstrated that myoepithelioma and PA form a uniform epigenetic class, supporting the theory of a single entity with a broad but continuous morphologic spectrum. Specific epigenetic signatures of BCA have, however, not been addressed in their study [13].

Therefore, in order to clarify the potential relationship between the abovementioned entities, we retrieved 30 cases of benign salivary gland tumors with morphological features of BCA and cellular PA from the authors’ registries, in which there were 17 cases of BCA with S100 protein–positive stroma, 7 cases of BCA without S100 protein–positive stromal cells, and 6 cases of myoepithelial cell–rich PA. We analyzed their histological, immunohistochemical, and genetic characteristics. To assess the applicability of DNA methylation–based tumor classification to our cohort, we analyzed 23 (of 30 cases) with high-quality DNA methylation profiles.

## Materials and methods

A retrospective search in the authors’ registries identified 30 cases of benign salivary gland tumors with morphological features of BCA and cellular PA, including 17 cases of BCA with an abundant S100 protein–positive stroma (group A). In addition, a control group of 7 cases of BCA without S 100 protein–positive spindle cell–rich stroma (group B) and 6 cases of cellular myoepithelial cell–rich PA (group C) was retrieved from the consultation files of the Tumor Registry at the Department of Pathology, Faculty of Medicine in Pilsen and Bioptic Laboratory, Ltd in Pilsen, Czech Republic, and tumor registries of the co-authors. All cases were reviewed by the senior author (AS) and two other head and neck pathologists (MB and GQ), and it was confirmed that they met the diagnostic criteria [1].

Clinical information of the cases was collected from hospital records and the referring pathologists. This study followed the rules of the institutional ethics review board of each participating institution, and the need to obtain informed consent was waived due to the retrospective nature of the analysis.

### Histological and immunohistochemical studies

For conventional microscopy, tissues were fixed in formalin, routinely processed, embedded in paraffin (FFPE), cut, and stained with hematoxylin and eosin. For immunohistochemistry, 4-μm-thick sections were cut from paraffin blocks and mounted on positively charged slides (TOMO, Matsunami Glass IND, Osaka, Japan). Sections were processed on a BenchMark ULTRA (Ventana Medical Systems, Tucson, AZ), deparaffinized, and subjected to heat-induced epitope retrieval by immersion in a CC1 solution (pH 8.6) at 95 °C. All primary antibodies used in this study are summarized in Table 1. Visualization was performed using the ultraView Universal DAB Detection Kit (Roche, Tucson, AZ) and ultraView Universal Alkaline Phosphatase Red Detection Kit (Roche, Tucson, AZ). The slides were counterstained with Mayer’s hematoxylin. Appropriate positive controls were employed. PLAG1 immunohistochemical examination was recognized as positive if moderate to strong nuclear staining was present at least focally (≥ 10% of tumor cells). Cytoplasmic, membranous, or non-specific diffuse granular staining was not considered positive. HMGA2 immunohistochemical stain was interpreted as positive only if strong diffuse nuclear staining was present.

**Table 1:** Antibodies used for immunohistochemical study

Antibody specificity		Clone	Dilution		Antigen retrieval/time	Source
AE1/3		AE1/AE3 + PCK26	RTU		EnVision High pH/30 min	Dako
CK7		OV-TL 12/30	1:800		EnVision High pH/30 min	Dako
CK14		SP53	1:800		EnVision High pH/30 min	Cell Marque
p63		DAK-p63	RTU		EnVision Low pH/30 min	Dako
p40		DAK-p40	RTU		EnVision High pH/30 min	Dako
SOX 10		SP267	RTU		CC1/64 min	Cell Marque
Ki-67		MIB-1	RTU		EnVision High pH/30 min	Dako
S100 protein		Polyclonal	RTU		EnVision High pH/30 min	Dako
LEF1		EPR2029Y	1:100		CC1/52 min	AbCam
Beta-catenin		14	RTU		CC1/64 min	Cell Marque
PLAG1		3B7	1:50		CC1/64 min	Merck
HMGA2		D1A7	1:100		CC1/64 min	Cell Signaling
EMA		E29	1:800		EnVision High pH/30 min	Dako
CK 5/6		D5/16 B4	1:200	EnVision High pH/30 min		Dako
CK 8	35betaH11		RTU	CC1/64 min		Cell Marque
CK 17	E3		RTU	CC1/64 min		Dako
CK18	DC10		RTU	CC1/64 min		Dako

*RTU*, ready to use; *CC1*, EDTA buffer pH 8.6 at 95 °C; EnVision High pH 9.0 at 97 °C; EnVision Low pH 6.0 at 97 °C; *min*, minutes

### Molecular studies

#### Nucleic acid extraction

DNA was extracted using the QIAsymphony DSP DNA Mini Kit (Qiagen, Hilden, Germany) and quantified using the Qubit BR DNA Assay Kit (Thermo Fisher Scientific, Waltham, MA). RNA was extracted using the Maxwell RSC DNA FFPE Kit and the Maxwell RSC Instrument (Promega, Madison, WI) according to the manufacturer’s instructions and quantified using the Qubit HS RNA Assay Kit (Thermo Fisher Scientific, Waltham, MA). The quality of DNA was assessed using the FFPE QC kit (Illumina, San Diego, CA), and the quality of RNA was assessed using the Agilent RNA ScreenTape Assay (Agilent, Santa Clara, CA). DNA samples with Cq < 5 and RNA samples with DV_200_ ≥ 20 were used for further analysis.

#### Illumina TruSight Oncology 500 Assay and RNA Pan-Cancer Panel

DNA and RNA libraries were prepared using the TruSight Oncology 500 Kit (Illumina, San Diego, CA) according to the manufacturer’s protocol, except for DNA enzymatic fragmentation using KAPA FragKit (KAPA Biosystems, Washington, MA). The original set of probes for fusion detection was replaced with TruSight RNA Pan-Cancer Panel (Illumina, San Diego, CA) targeting 1385 genes. The complete list of genes can be found on the manufacturer’s websites: https://www.illumina.com/content/dam/illumina-marketing/documents/products/gene_lists/gene_list_trusight_oncology_500.xlsx and https://emea.illumina.com/content/dam/illumina-marketing/documents/products/gene_lists/gene_list_trusight_pan_cancer.xlsx. Data analysis for fusion detection was performed using Dragen RNA app version 4.0.4 (Illumina, San Diego, CA). The number of unique mapped reads was set at 18 million, and minimal fusion split reads at 5. DNA panel analysis was performed using TruSight Oncology 500 Local app version 2.2.0.12, including TMB analysis (set for TMB high > 10 mut/Mb) and CNV analysis — amplification of 59 genes. Variant filtering and annotation were performed using the OmnomicsNGS software (Euformatics, Espoo, Finland). A custom variant filter was set up, including only non-synonymous variants with coding consequences, read depth greater than 50, and allele frequency (AF) > 5%. Benign variants according to the ClinVar database [14] were also excluded. The remaining subset of variants was checked visually, and suspected artefactual variants were excluded.

#### Fluorescence in situ hybridization (FISH) analysis of PLAG1 gene

Prior to FISH, hematoxylin and eosin–stained slides were examined to determine the areas for cell evaluation. Then, a 4-µm-thick FFPE section was placed onto a positively charged slide. The unstained slide was routinely deparaffinized and incubated in 1 × Target Retrieval Solution Citrate pH 6 (Dako, Glostrup, Denmark) for 40 min at 95 °C, subsequently cooled for 20 min at room temperature in the same solution and washed in deionized water for 5 min. The slide was digested in protease solution with pepsin (0.5 mg/mL) (Sigma-Aldrich, St Louis, MO, USA) in 0.01 M HCl at 37 °C for 45 to 60 min, depending on sample conditions. The slide was then rinsed in deionized water for 5 min, dehydrated in a series of ethanol solutions (70%, 85%, and 96% for 2 min each), and air-dried.

For rearrangements involving *PLAG1* gene, SureFISH PLAG1 5′ BA 625 kb and SureFISH PLAG1 3′ BA 295 kb were used (SureFish/Agilent Technologies). FISH analysis was performed and interpreted as described elsewhere [15].

#### Methylation analysis

Methylation analysis was performed using Infinium Methylation EPIC BeadChip kit (Illumina, San Diego, CA) with over 850,000 methylation sites. Bisulfite conversion of DNA was carried out using EZ DNA Methylation-Direct Kit (Zymo Research, Irvine, CA) followed by DNA restoration using Infinium HD FFPE DNA Restore Kit (Illumina, San Diego, CA). Methylation chip was prepared using Infinium Methylation EPIC BeadChip kit v2 (Illumina, San Diego, CA) according to the manufacturer’s protocol and then scanned on NextSeq 550 (Illumina, San Diego, CA). Quality control on methylation data was performed using BeadArray Controls Reporter software.

The EPICv2 idat files were processed using the pipeline outlined in EPICv2 Workflow: From idats to DMRs — using DMRcate to identify Differentially Methylated Regions from EPICv2 data (https://clark-lab.github.io/EPICv2_tutorial/). The beta values were filtered to select the top 2000 most variable probes based on variance. This subset of probes was then employed for 2D visualization of samples using the UMAP clustering algorithm and heatmap representation of the beta value profiles.

## Results

### Histopathological and immunohistochemical findings

#### Basal cell adenoma with S100 protein–positive stroma (group A)

The cohort included 17 cases of BCA with S100 protein–positive stromal cells. Among these, 7 patients were male and 10 were female, with an average age of 62 years (age range, 43–74 years). All cases were located in the parotid gland. Clinical, immunohistochemical, and molecular genetic results are described in Table 2.

**Table 2:** Clinical and immunohistochemical findings

Pathological unit	Basal cell adenoma with S-100 protein–positive stroma	Basal cell adenoma without S-100 protein–positive stroma	Pleomorphic adenoma (cellular and oncocytic)
**Clinical data**	***N* = 17**	***N* = 7**	***N* = 6**
**• Age (range) years**	62 (43–74)	50 (22–64)	60 (44–73)
**• Sex male/female**	7/10	1/6	2/4
**• Site**	• Parotid (*n* = 17)	• Parotid (*n* = 5) • Palate (*n* = 1) • Buccal mucosa (*n* = 1)	• Parotid (*n* = 6)
**Immunohistochemistry**			
**• S100 protein** **•** Positive **•** Negative/not done	17 0/0	7 0/0	4 2/0
**• SOX10** **•** Positive **•** Negative/not done	17 0/0	7 0/0	5 1/0
**• HMGA2** **•** Positive **•** Negative/not done	1 10/6	2 3/2	1 4/1
**• PLAG1** **•** Positive **•** Negative/not done	0 11/6	0 4/3	4 2/0
**• LEF1** **•** Positive **•** Negative/not done	17 0/0	1 3/3	0 0/6
**• BETA-catenin** **•** Nuclear positivity **•** Negative/not done	15 2/0	2 2/3	0 0/6
**• p63/p40** **•** Biphasic positivity **•** Negative/not done	17 0/0	4 3/0	5 1/0

**Fig. 1:**
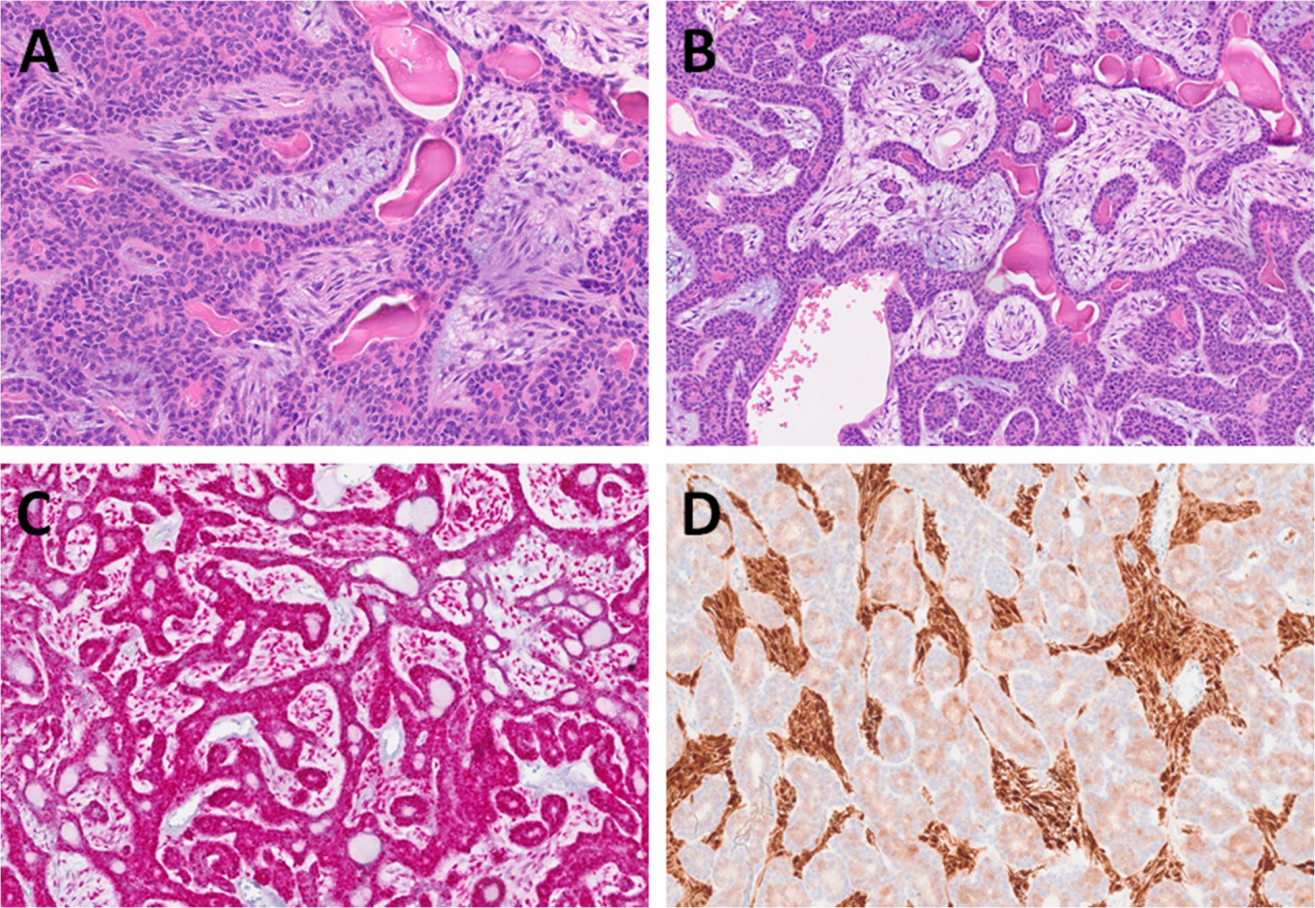
BCAs with a myoepithelial cell–rich stroma (group A) were biphasic tumors with tubular, trabecular, or cribriform arrangements and peripheral palisading of tumor cells (**A**). The stroma consisted of dispersed spindle-shaped or triangular cells (**B**). SOX10 was positive in both the epithelial and stromal components (**C**), while S100 protein was positive in the stromal component (**D**)

The tumors were well-circumscribed or encapsulated by a thick fibrous capsule. Histologically, they displayed a biphasic pattern, comprising tubular, trabecular, or cribriform arrangements with peripheral palisading of tumor cells (Fig. 1A). The tumor nuclei were round to oval, without atypia or mitotic activity. The cytoplasm was basophilic. Two cases exhibited multiple distinct unencapsulated nodules. The stroma varied in prominence, showing hyalinization with occasional minor myxoid changes and containing dispersed spindle-shaped or triangular cells (Fig. 1B).

SOX10 immunoreactivity was detected in both the epithelial (luminal and abluminal) and stromal components. In the luminal component, SOX10 was positive in 13 out of 17 cases. In the abluminal epithelial component, SOX10 expression was observed in all cases tested (17/17). In the stromal component, SOX10 was positive in 16 out of 17 cases **(**Fig. 1C). S100 protein was strongly positive in all tested cases within the stromal component (17/17). In the epithelial components, S100 expression was focal and limited to weak cytoplasmic staining. In the luminal component, S100 was positive in 13 out of 17 cases, while in the abluminal epithelial component, it was observed in 3 out of 17 cases (Fig. 1D).

**Fig. 2:**
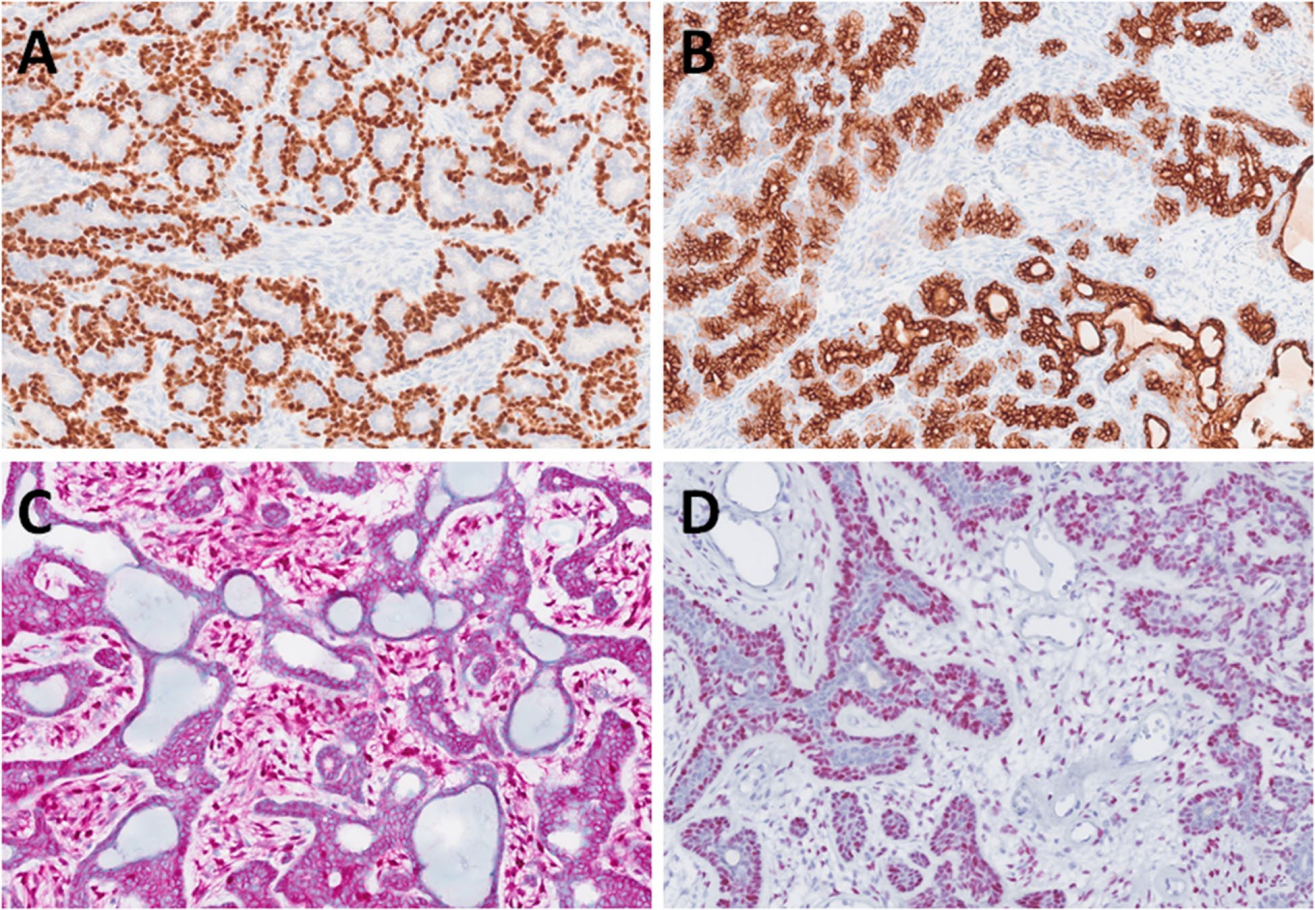
p63 was expressed in the myoepithelial layer of tumor cells, highlighting the biphasic pattern (**A**), while cytokeratin marked the luminal cells (**B**). Nuclear beta-catenin expression was observed in both epithelial and stromal myoepithelial-like cells, with more pronounced expression in the latter component (**C**). LEF1 showed abluminal positivity in the epithelial component and in the stromal cells (**D**)

In the luminal component, both p63 and p40 were negative in all tested cases (0/17). In the abluminal epithelial component, diffuse nuclear positivity for p63 and p40 was observed in all cases (17/17) (Fig. 2A). In the stromal component, p63 was focally positive in 1 out of 17 cases, while p40 remained negative in all tested cases (0/17). A strong and diffuse staining was observed in the luminal component for AE1/AE3 (5/5 cases tested), CK7 (4/4), CK8 (7/7), CK18 (7/7), CK17 (5/7), and CK14 (5/6) (Fig. 2B).

In the abluminal epithelial component, AE1/AE3 was positive in 5/5 cases tested, although with weaker staining intensity compared to luminal cells. CK14 was expressed in 5 out of 6 cases analyzed. In positive cases, CK14 expression in the abluminal component was stronger and more diffuse compared to the weak and focal positivity observed in the luminal component. In the abluminal component, CK17 was positive in 6 out of 7 cases investigated, and CK18 in 2 out of 7 cases, whereas CK7 and CK8 were consistently negative (0/4 and 0/7 cases assessed, respectively).

Comparatively, CK7, CK8, and CK18 were more frequently expressed in the luminal component, with CK7 being positive in 4/4 cases and CK8 and CK18 in 7/7 cases each, compared to 0/4, 0/7, and 2/7 positive tested cases, respectively, in the abluminal epithelial component. Conversely, CK17 showed a slightly higher expression in the abluminal epithelial component (6/7 cases tested) than in the luminal component (5/7 cases tested).

In the stromal component, no expression of AE1/AE3, CK7, CK8, CK17, or CK18 was detected in any of the cases tested. EMA expression was detected in all tested cases within the luminal component (5/5 cases). In contrast, EMA was negative in both the abluminal epithelial (0/5 cases tested) and stromal components (0/5).

Nuclear β-catenin expression was observed in both the epithelial and stromal cells, with variable frequency among the cases. In the luminal component, β-catenin nuclear positivity was detected in 1 out of 17 cases. In the abluminal epithelial component, nuclear β-catenin positivity was observed in 15 out of 17 cases. Among the remaining two cases, one was completely negative, while the other showed cytoplasmic staining without nuclear localization. Similarly, in the stromal component, nuclear β-catenin expression was observed in 15 out of 17 cases. In most cases, spindle-shaped “stromal” cells were more commonly positive than luminal cells (Fig. 2C). LEF1 was expressed in an abluminal pattern within the epithelial component, with nuclear positivity observed in all tested cases (17/17). In the luminal component, LEF1 expression was absent (0/17 cases). In the stromal component, LEF1 nuclear positivity was also consistently observed in all cases tested (17/17) (Fig. 2D). HMGA2 expression was observed in one case (1/11 cases tested). In contrast, PLAG1 was negative in all cases tested (0/11), with no nuclear staining detected in either epithelial or stromal components.

## Basal cell adenoma without S100 protein–positive stromal cells (group B)

The first control cohort of BCA without S100 protein–positive stromal cells included one male and six female patients, with an average age of 50 years (age range, 22–64 years). The parotid gland was the primary site of origin in four cases, while one case was located in the palate and one case in the buccal mucosa. Clinical, immunohistochemical, and molecular genetic results are summarized in Table 2.

**Fig. 3:**
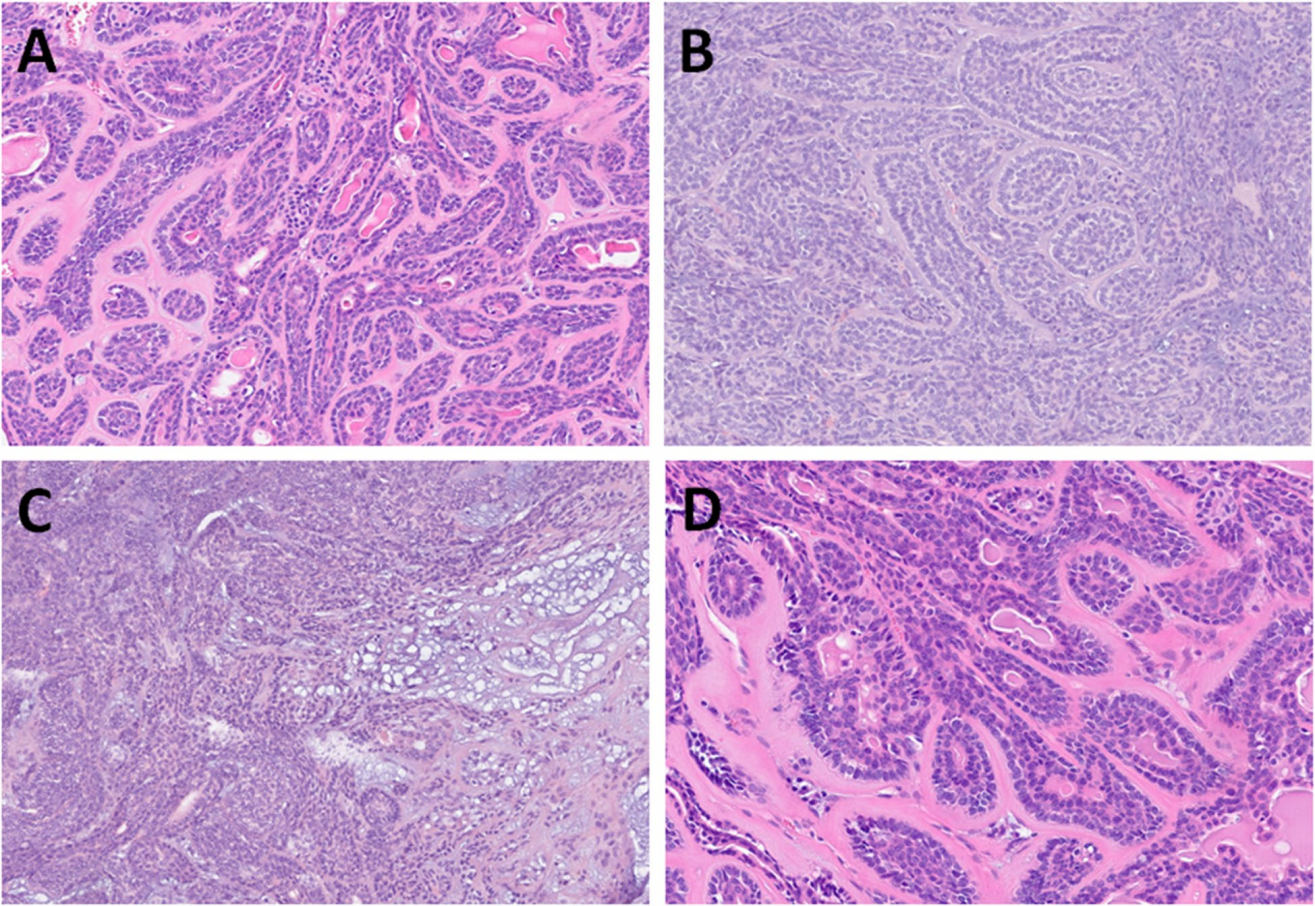
BCAs without myoepithelial cell–rich stroma (group B) were of similar architecture to group A, composed of tubular, trabecular, or cribriform (**A**) but with minimal intervening stroma (**B**). Small vascular spaces or occasional myxoid changes were observed in the spaces between the trabeculae (**C**). Thickened basement membranes surrounded some tubules in some cases (**D**)

**Fig. 4:**
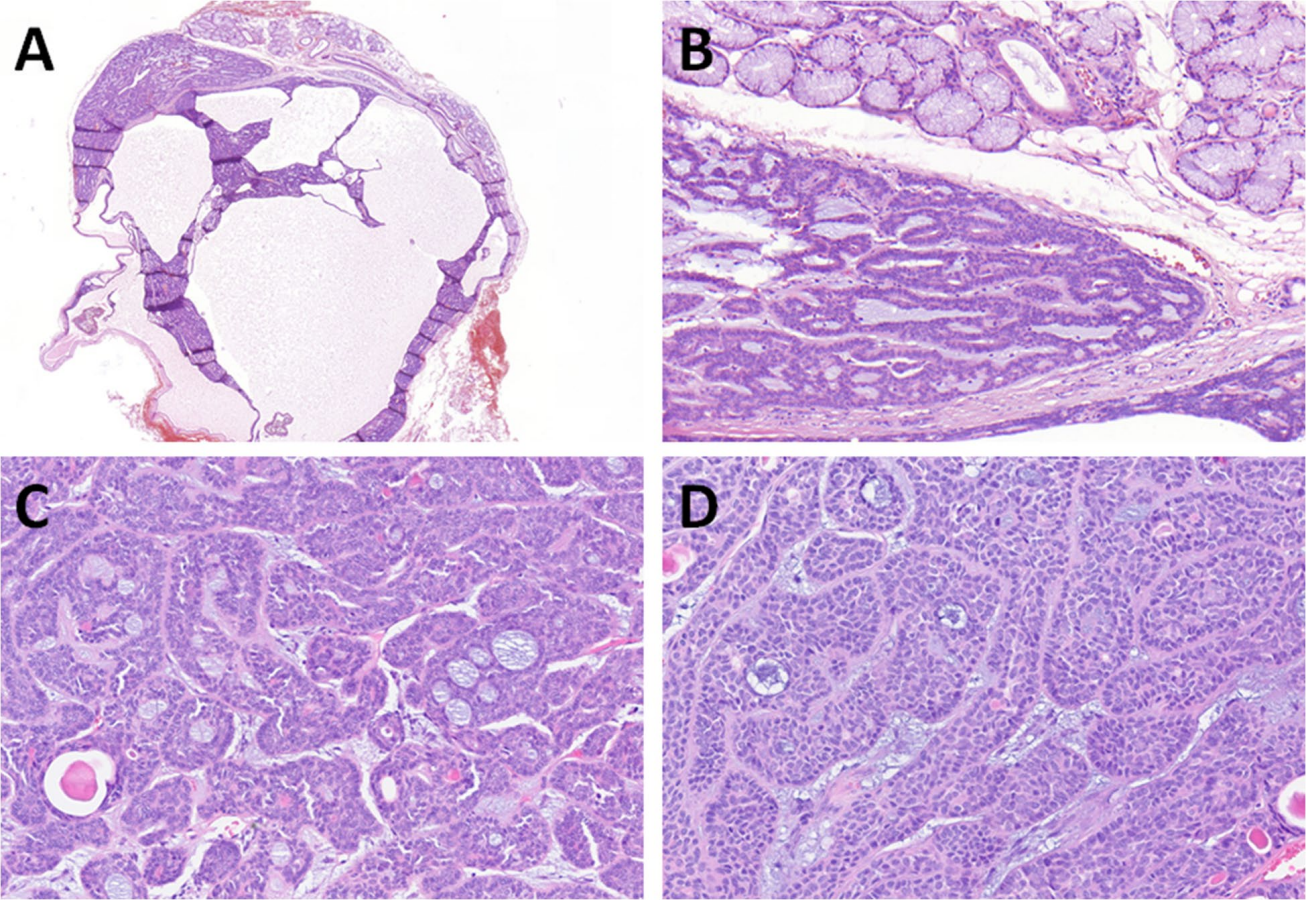
One case was cystic BCA arising from the minor salivary gland of buccal mucosa (**A**). Large cystic spaces compressed the remaining tumor tissue toward the periphery of the capsule (**B**). Cribriform growth pattern (**C**) and nuclear palisading were common features (**D**)

All tumors were well-circumscribed and encapsulated. They exhibited tubular, trabecular, or cribriform architecture, with compact tubules and trabeculae and minimal intervening stroma (Fig. 3A, B). Focally, small vascular lumina or occasional myxoid changes were observed in the spaces between the trabeculae (Fig. 3C). In scattered areas, thickened basement membranes surrounded some tubules (Fig. 3D). In one case of cystic BCA arising from the minor salivary gland of buccal mucosa (Fig. 4A), large cystic spaces compressed the remaining tumor tissue toward the periphery of the capsule (Fig. 4B), while in other cases, the cribriform growth pattern (Fig. 4C) and nuclear palisading were common features (Fig. 4D).

**Fig. 5:**
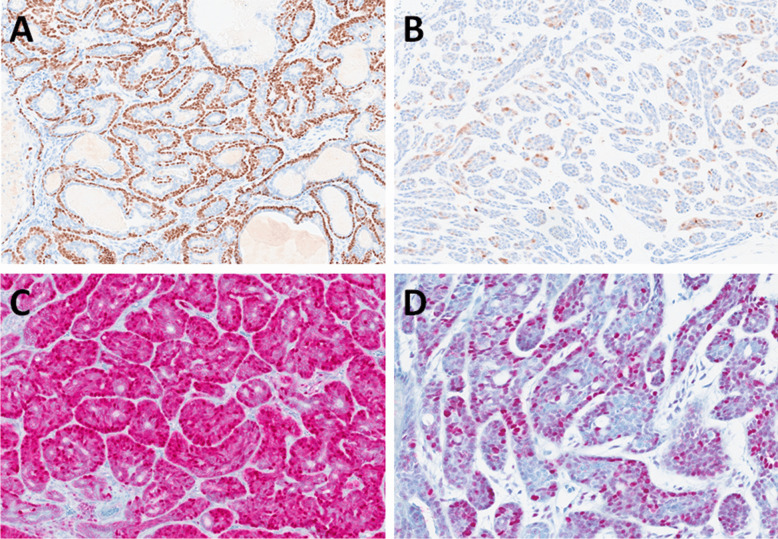
p63 was positive in an abluminal layer in four cases (**A**). S100 protein showed a weak and focal cytoplasmic staining pattern in epithelial cells, while stromal cells were mostly negative (**B**). Two cases showed focal nuclear expression of beta-catenin (**C**). LEF1 nuclear staining was detected in one case with an accentuated positivity of the epithelial cells at the periphery of the tumor nests (**D**)

The myoepithelial markers p63 and p40 were positive in the abluminal layer in four cases (Fig. 5A), while three cases were negative. HMGA2 was positive in 2 out of 5 tested cases, and PLAG1 was negative in all tested cases (*n* = 4). SOX10 was positive in all cases. S100 protein exhibited a weak and focal cytoplasmic staining pattern in epithelial cells, while the scattered stromal component was either negative or showed a maximum of 5% of positive cells (Fig. 5B). We tested four cases for β-catenin; two showed focal nuclear expression (Fig. 5C) while two showed only weak cytoplasmic positivity. LEF1 nuclear staining was detected in one case with an accentuated positivity of the epithelial cells at the periphery of the tumor nests (Fig. 5D).

## Myoepithelial cell–rich pleomorphic adenoma (group C)

The second control cohort of cellular PAs consisted of four females and two males, with an average age of 60 years (range, 44–73 years). All these cases were localized in the parotid gland. Two cases were dominantly oncocytic PAs, and four were cellular spindle-shaped cell-rich PAs with scattered chondromyxoid components. The tumors were multinodular and circumscribed or encapsulated and had a bosselated surface.

**Fig. 6:**
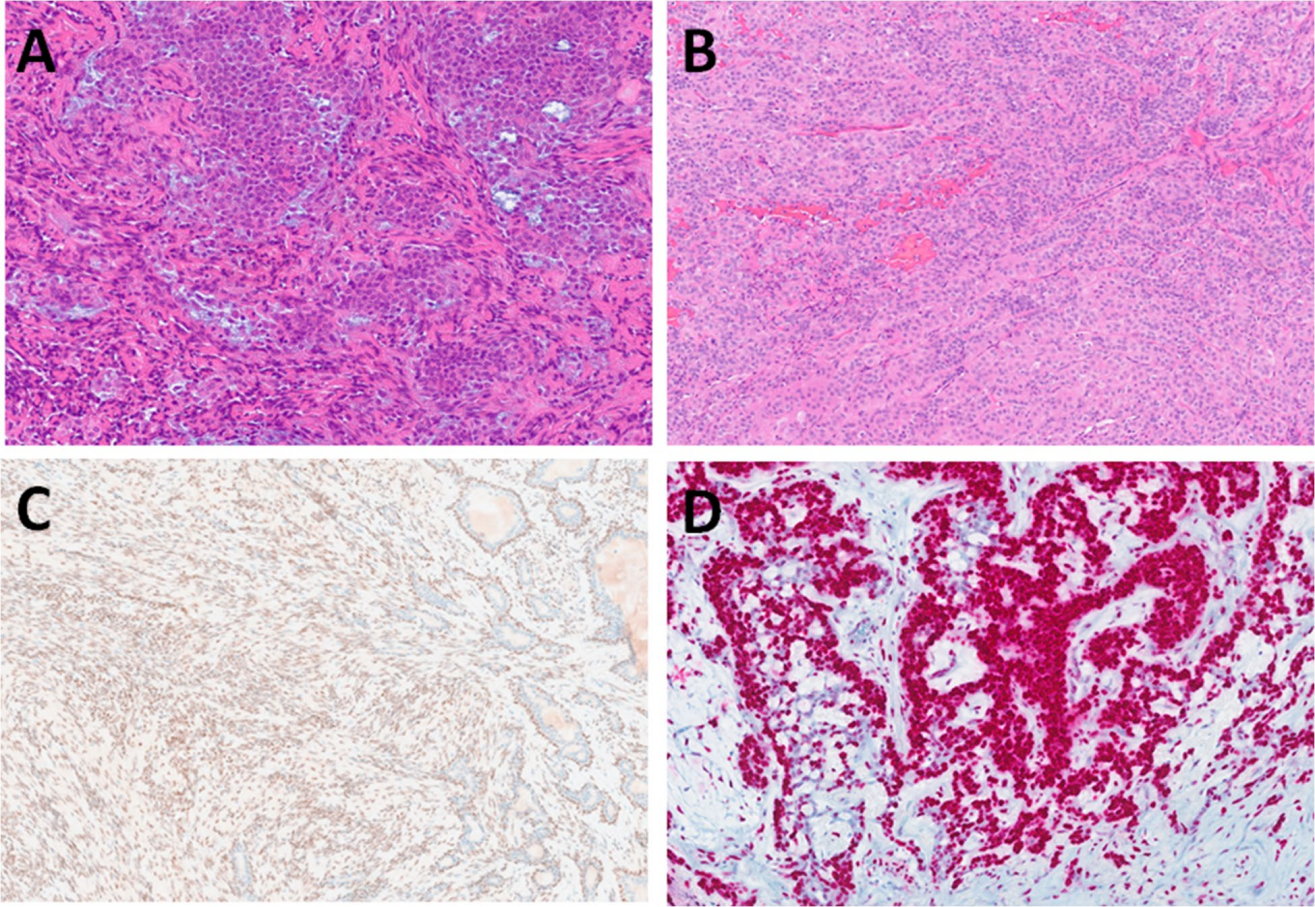
Group C consisted of cellular PAs with tubular, trabecular, or solid morphology with a limited amount of hyalinized and/or myxoid stroma (**A**) and oncocytic PA mostly of solid growth with minimal myxoid stromal changes (**B**). Both oncocytic PAs were positive for PLAG1 (**C**). One case of cellular PA was positive for HMGA2 (**D**)

Cellular PAs were tubular, trabecular, or solid masses with a limited amount of hyalinized and/or myxoid stroma (Fig. 6A). Tumor cells were plasmacytoid, spindled, or round with bland round to oval nuclei with a minimal nuclear pleomorphism. Two cases were of oncocytic morphology, and both showed only focal expression of p40 and p63 in the abluminal layer, specifically in areas with non-oncocytic morphology (Fig. 6B). They were strongly and diffusely positive for SOX10 and S100 protein, and both expressed the PLAG1 marker (Fig. 6C). Of the four remaining cellular PAs, three were positive for p63 and p40, while one was negative. Two cases of PA were positive for S100 protein, while one showed only weak expression, and three were negative. Three cases of PA were strongly positive for SOX10, while two were not stained. Two cases were positive for PLAG1 protein, and one case was positive for HMGA2 protein **(**Fig. 6D**)**, while one case was negative for both markers.

### Findings in next-generation sequencing (NGS) and fluorescence in situ hybridization

The molecular genetic results are summarized in Table 3. Eleven cases of BCA with S100 protein–positive stromal cells were characterized by one or more pathogenic or likely pathogenic mutations. These included, especially, the *CTNNB1* mutation detected in 11 cases. Except for that, mutations of *ETV5*, *FOXL2*, *NBN*, *CHEK2*, *MUTYH*, and *TSHR* were found, the latter three suspiciously germline. Three cases were negative for *CTNNB1* and any other detectable mutation, and 3 were not done or analyzable. *PLAG1* FISH testing yielded no signs of gene rearrangement in 15 cases, while one case was tested positive for rearrangement and one case was not analyzable.

**Table 3:** Genetic results of NGS, FISH, and methylation analysis

Case	Group	Reclassification	TS500D variants P/LP	TS500D VUS	TS500P	PLAG1 FISH
1	A	A	CTNNB1 c.104 T > C, p.(Ile35Thr), (alias I35T), AF: 37%, (NM_001904.4, NP_001895.1, chr3:41,266,107, hg19); ETV5 c.1209 + 1G > A, AF: 6%, (NM_004454.3, chr3:185,774,863, hg19); MUTYH c.1178G > A, p.(Gly393Asp), (alias G393D), AF: 45% — susp. germ., (NM_001048174.2, chr1:45,797,228, hg19)	FGFR1 c.1585A > G, p.(Ile529Val), (alias I529V), AF: 7%, (NM_023110.3, chr8:38,275,448, hg19); PMS1 c.174_175delinsTA, p.(Glu59Lys), (alias E59K), AF: 40% — susp. germ., (NM_000534.5, chr2:190,660,536, hg19)	ND	neg
2	A	A	CTNNB1 c.104 T > C, p.(Ile35Thr), (alias I35T), AF: 25%, (NM_001904.4, NP_001895.1, chr3:41,266,107, hg19)	BCOR c.4934C > G, p.(Pro1645Arg), (alias P1645R), AF: 50% — susp. germ., (NM_001123385.2, NP_001116857.1, chrX:39,913,181, hg19); EPCAM c.605A > C, p.(Lys202Thr), (alias K202T), AF: 49% — susp. germ., (NM_002354.3, NP_002345.2, chr2:47,606,141, hg19); KMT2C c.2516G > T, p.(Arg839Ile), (alias R839I), AF: 15%, (NM_170606.3, NP_733751.2, chr7:151,945,003, hg19); LATS2 c.686_691del, p.(Gln229_His230del), (alias Q229_H230del), AF: 46%, susp. germ., (NM_014572.3, NP_055387.2, chr13:21,563,227, hg19)	ND	neg
3	A	A	CTNNB1 c.104 T > C, p.(Ile35Thr), (alias I35T), AF: 8%, (NM_001904.4, NP_001895.1, chr3:41,266,107, hg19); CHEK2 c.599 T > C, p.(Ile200Thr), (alias I200T/I157T), AF: 44% — susp. germ., (NM_001005735.2, NP_001005735.1, chr22:29,121,087, hg19); MUTYH c.1178G > A, p.(Gly393Asp), (alias G393D), AF: 52% — susp. germ., (NM_012222.3, NP_036354.1, chr1:45,797,228, hg19)	RASA1 c.418_435del, p.(Pro140_Pro145del), (alias P140Pdel), AF: 49% — susp. germ., (NM_002890.3, NP_002881.1, chr5:86,564,676, hg19); TET1 c.3053_3055delinsGTG, p.(Asn1018_Lys1019delinsSerGlu), (alias N1018_K1019delinsSE), AF: 47% — susp. germ., (NM_030625.3, NP_085128.2, NP_085128.2, chr10:70,405,539, hg19)	ND	neg
4	A	NA	ND	ND	ND	NA
5	A	A	CTNNB1 c.104 T > C, p.(Ile35Thr), (alias I35T), AF: 37%, (NM_001904.4, NP_001895.1, chr3:41,266,107, hg19)	IGF2 c.604C > T, p.Leu202Phe, (alias L202F), AF: 45% — susp. germ., (NM_000612.6, chr11:2,154,324, hg19); LRP1B c.9728C > T, p.(Thr3243Ile), (alias T3243I), AF: 51% — susp. germ., (NM_018557.3, chr2:141,215,118, hg19)	ND	neg
6	A	A	CTNNB1 c.104 T > C, p.(Ile35Thr), (alias I35T), AF: 48%, (NM_001904.4, NP_001895.1, chr3:41,266,107, hg19); TSHR c.104 T > C, p.(Ile35Thr), (alias I35T), AF: 40% — susp. germ. (NM_000369.5chr3:41,266,107, hg19)	neg	ND	neg
7	A	A	neg	ABL1 c.659A > T, p.(His220Leu), (alias H220L), AF: 9%, (NM_005157.6, chr9:133,738,202, hg19); DNMT1 c.1703G > A, p.(Arg568Gln), (alias R568Q), AF: 51% — susp. germ., (NM_01130823.3, chr19:10,265,391, hg19); ZFHX3 c.7850A > C, p.(Lys2617Thr), (alias K2617T), AF: 49% — susp. germ., (NM_ 006885.4, chr16:72,828,731, hg19)	ND	neg
8	A	A	CTNNB1 c.104 T > C, p.(Ile35Thr), (alias I35T), AF: 22%, (NM_001904.4, NP_001895.1, chr3:41,266,107, hg19); FOXL2 c.226A > G, p.(Thr76Ala), (alias T76A), AF: 27%, (NM_023067.4, chr3:138,665,339, hg19); NBN c.142_143insAT, p.(Leu48TyrfsTer2), (alias L48Y*X2), AF: 25%, (NM_002485.5, chr8:90,994,978, hg19)	no	ND	neg
9	A	C	NA	NA	ND	pos*
10	A	A	neg	NCOR1 c.3356C > G, p.(Pro1119Arg), (alias P1119R), AF: 47% — susp. germ., (NM_006311.4, NP_006302.2, chr17:15,983,766, hg19)	ND	neg
11	A	A	neg	neg	neg	neg
12	B	NA	ND	ND	ND	NA
13	A	A	CTNNB1 c.104 T > C, p.(Ile35Thr), (alias I35T), AF: 32%, (NM_001904.4, NP_001895.1, chr3:41,266,107, hg19)	neg	neg	neg
14	A	NA	ND	ND	neg	neg
15	B	B	neg	KAT6A c.1591 T > C, p.(Tyr531His), (alias Y531H), AF: 44% — susp. germ., (NM_006766.5, NP_006757.2, chr8:41,812,821, hg19)	neg	neg
16	A	A	CTNNB1 c.104 T > C, p.(Ile35Thr), (alias I35T), AF: 45%, (NM_001904.4, NP_001895.1, chr3:41,266,107, hg19)	AXIN1 c.1118G > A, p.(Arg373His), (alias R373H), AF: 42% — susp. germ., (NM_003502.4, NP_003493.1, chr16:354,440, hg19); CDH1 c.1016_1027del, p.(Pro339_Thr342del), (alias P339_T342del), AF: 47% — susp. germ., (NM_004360.5, NP_004351.1, chr16:68,846,042, hg19); ZFHX3 c.8551G > T, p.(Gly2851Cys), (alias G2851C), AF: 48% — susp. germ., (NM_006885.4, NP_008816.3, chr16:72,828,030, hg19)	neg	neg
17	A	A	CTNNB1 c.104 T > C, p.(Ile35Thr), (alias I35T), AF: 46%, (NM_001904.4, NP_001895.1, chr3:41,266,107, hg19)	CCND3 c.774_775delinsTG, p.(Ser259Ala), (alias S259A), AF: 47% — susp. germ., (NM_001760.5, NP_001751.1, chr6:41,903,782, hg19)	neg	neg
18	A	A	CTNNB1 c.104 T > C, p.(Ile35Thr), (alias I35T), AF: 20%, (NM_001904.4, NP_001895.1, chr3:41,266,107, hg19)	FGFR4 c.2098 T > C, p.(Ser700Pro), (alias S700P), AF: 46% — susp. germ., (NM_213647.3, NP_998812.1, chr5:176,523,687, hg19); LZTR1 c.824G > A, p.(Arg275Gln), (alias R275Q), AF: 43% — susp. germ., (NM_006767.4, NP_006758.2, chr22:21,345,949, hg19); MSH6 c.3600A > G, p.(Ile1200Met), (alias I1200M), AF: 35%, (NM_000179.3, NP_000170.1, chr2:48,032,800, hg19); SMARCB1 c.1028C > T, p.(Thr343Met), (alias T343M), AF: 45% — susp. germ., (NM_003073.5, NP_003064.2, chr22:24,175,800, hg19)	neg	neg
19	A	A	CTNNB1 c.104 T > C, p.(Ile35Thr), (alias I35T), AF: 49%, (NM_001904.4, NP_001895.1, chr3:41,266,107, hg19)	SH2B3 c.1454_1477del, p.(Asp485_Trp492del), (alias D485_W492del), AF: 39%, (NM_005475.3, NP_005466.1, chr12:111,885,815, hg19)	neg	neg
20	NA possibly C	NA	NA	NA	ND	NA
21	B	B	TET2 c.697del, p.(Tyr233IlefsTer17), (alias Y233I*X17), AF: 44% — susp. germ., (NM_017628.4, NP_060098.3, chr4:106,155,795, hg19)	APC c.6124 T > C, p.(Cys2042Arg), (alias C2042R), AF: 44% — susp. germ., (NM_000038.6, NP_000029.2, chr5:112,177,415, hg19); TP63 c.292A > C, p.(Met98Leu), (alias M98L), AF: 52% — susp. germ., (NM_003722.5, NP_003713.3, chr3:189,456,531, hg19)	neg	neg
22	B	B	neg	neg	neg	neg
23	B	A	CTNNB1 c.104 T > C, p.(Ile35Thr), (alias I35T), AF: 43%, (NM_001904.4, NP_001895.1, chr3:41,266,107, hg19); LRP1B c.13416-1G > T, AF: 42% — susp. germ., (NM_018557.3, chr2:140,995,866, hg19)	POLE c.407A > T, p.(Lys136Ile), (alias K136I), AF: 47% — susp. germ., (NM_006231.4, NP_006222.2, chr12:133,256,556, hg19)	neg	neg
24	B	A	CTNNB1 c.104 T > C, p.(Ile35Thr), (alias I35T), AF: 43%, (NM_001904.4, NP_001895.1, chr3:41,266,107, hg19); NUP93 c.2349 + 1G > T, AF: 44% — susp. germ., (NM_014669.5, chr16:56,875,746, hg19)	CHEK2 c.246_260del, p.(Asp82_Glu86del), (alias D82_E86del), AF: 41% — susp. germ., (NM_001005735.2, NP_001005735.1, chr22:29,130,449, hg19)	neg	neg
25	C	C	neg	AKT1 c.328C > A, p.(Leu110Ile), (alias L110I), AF: 46% — susp. germ., (NM_005163.2, NP_005154.2, chr14:105,242,096, hg19); BAP1 c.1138G > T, p.(Gly380Cys), (alias G380C), AF: 46% — susp. germ., (NM_004656.4, NP_004647.1, chr3:52,438,581, hg19); ROS1 c.5776C > T, p.(His1926Tyr), alias H1926Y), AF: 47% — susp. germ., (chr6:117,642,423, hg19)	neg	pos
26	C	C	neg	BMPR1A c.1245A > C, p.(Glu415Asp), (alias E415D), AF: 51% — susp. germ., (NM_004329.3, NP_004320.2, chr10:88,681,355, hg19)	neg	pos
27	C	C	BCOR c.3848-6_3850del, AF: 7%, (NM_001123385.2, chrX:39,922,321, hg19)	JAK3 c.23C > T, p.(Thr8Met), (alias T8M), AF: 48% — susp. germ., (NM_000215.4, NP_000206.2, chr19:17,955,204, hg19); KDR c.3695G > C, p.(Arg1232Pro), (alias R1232P), AF: 44% — susp. germ., (NM_002253.3, NP_002244.1, chr4:55,948,770, hg19)	MEG3::PLAG1, exon 1::exon 3, unknown, NR_003530.1, NM_002655.3, chr14:101,292,554, chr8:57,083,748, Hg19	pos
28	C	C	neg	AR c.10C > A, p.(Gln4Lys), (alias Q4K), AF: 46% — susp. germ., (NM_00044.6, chrX:66,764,998, hg19); BRCA2 c.4798_4800del, p.(Asn1600del), (alias N1600del), AF: 9%, (NM_000059.4, NP_000050.3, chr13:32,913,286, hg19); JAK2 c.683G > A, p.(Arg228Gln), (alias R228Q), AF: 45% — susp. germ., (NM_001322194.2, NP_001309123.1, chr9:5,054,631, hg19)	ACTA2::PLAG1, exon 1::exon 3,, NM_001613.4, NM_002655.3, chr10:90,712,488, chr8:57,083,748, Hg19	pos
29	B	B	neg	neg	neg	neg
30	C	C	DNMT3A c.1271del, p.(Pro424HisfsTer227), (alias P424H*X227), AF: 7%, (NM_022552.5, NP_072046.2, chr2:25,469,496, hg19)	PIK3CG c.1222_1240delinsATA, p.(Val408Ilefs*17), (alias V1408I*X17), AF: 44% — susp. germ., (NM_002649.3, NP_002640.2, chr7:106,509,228, hg19)	ND	neg**

^*^Atypical FISH pattern

^**^HMGA2 IHC positive

*susp. germ.*, suspectly germline, allele frequency (AF) ≥ 40% (except *CTNNB1* mutation); *neg*, negative; *pos*, positive; *ND*, not done; *NA*, not analyzable

In three cases of BCA without S100 protein–positive stroma, NGS identified one or more gene mutations, including *CTNNB1* mutation in two cases and several suspected germline alterations in genes *LRP1B*, *NUP93*, and a *TET2*. The remaining two cases were negative for any mutations. All cases were negative for *PLAG1* gene break by FISH, except for one non-analyzable case.

In the third group including oncocytic and cellular PAs, NGS detected *BCOR* and *DNMT3A* gene mutations and *MEG3::PLAG1* fusion (exon 1::exon 3) and *ACTA2::PLAG1* fusion (exon 1::exon 3). Four cases were positive for *PLAG1* gene break by FISH, while one case was negative. The latter case was positive for *HMGA2* protein by immunohistochemistry (IHC). One case was not analyzable by FISH.

### Methylation analysis and molecular classification

**Fig. 7:**
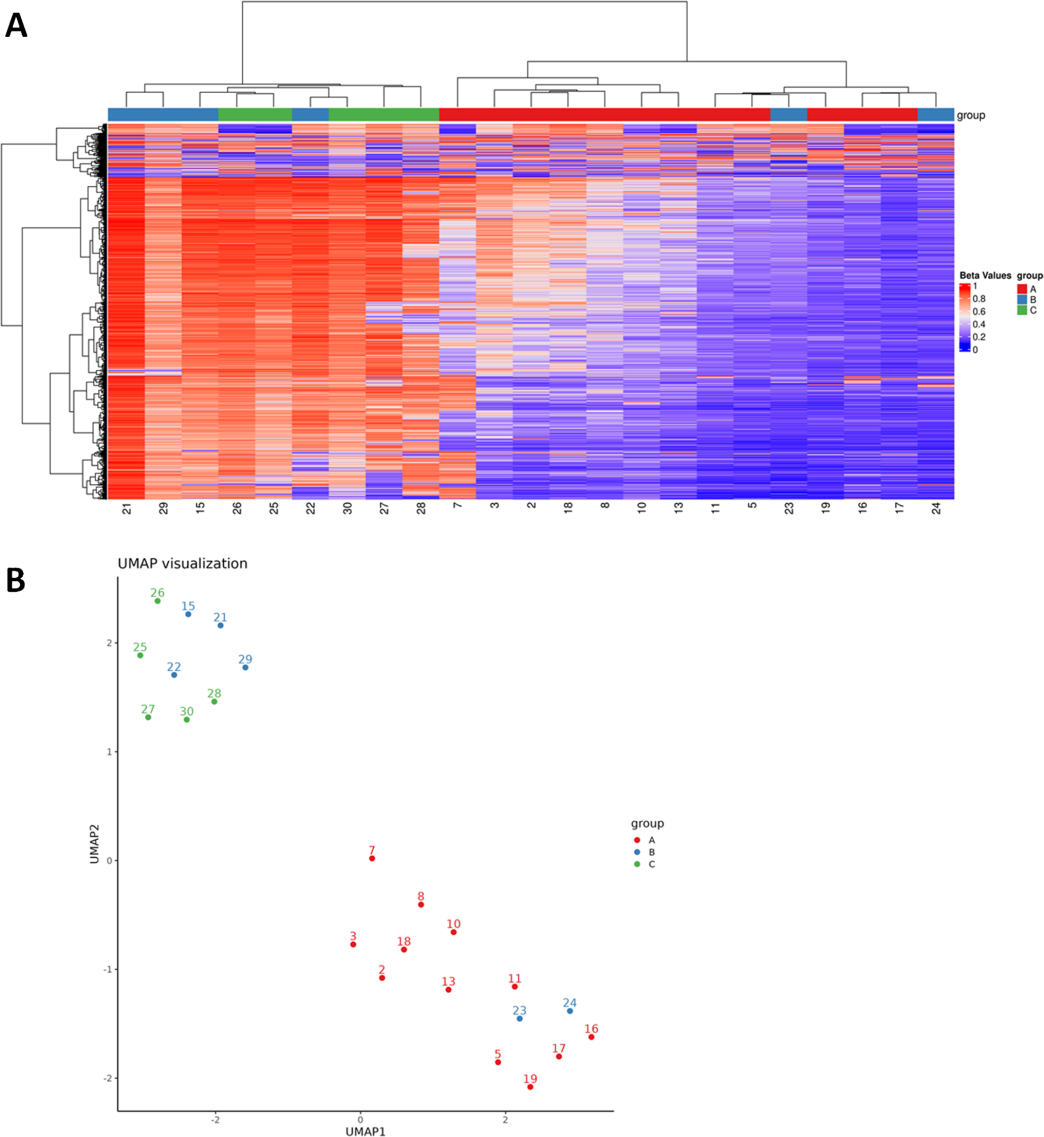
Heatmap representation of methylation profile clusters (**A**) and UMAP 2D visualization (**B**)

Among the 23 tumors with informative methylation profiles, unsupervised clustering resolved two well-defined molecular clusters (Fig. 7A, B).

Cluster 1 comprised all 12 BCA cases with S100-positive stromal cells (pre-analytic *group A*) together with two tumors that had originally been assigned to *group B* (cases 23 and 24). Both of these reassigned tumors harbored activating CTNNB1 mutations (Table 3) and, on histologic review, exhibited focal S100-positive stroma, supporting their re-classification into group A.

Cluster 2 contained the remaining four BCA cases without S100-positive stroma from *group B* plus all five pleomorphic adenomas (*group C*). Within this cluster, case 9, initially categorized as group A, showed a *PLAG1* break by FISH and was therefore reassigned to group C.

## Discussion

Tumors of the major and minor salivary glands histologically encompass a diverse and often overlapping spectrum of diagnostically challenging neoplasms. Despite recent advances in molecular testing and the identification of tumor-specific mutations or gene fusions, there remains a need to clarify certain aspects of the taxonomy of tumors with overlapping features. We have recently encountered such an example of a cohort of well-circumscribed salivary neoplasms that exhibited a basaloid appearance. These tumors were characterized by solid, trabecular, and tubular growth patterns similar to conventional BCA, and were associated with abundant cellular “stroma” composed of spindle-shaped S100 protein–positive cells. Contrary to the terminology, conventional BCA does not consist solely of basaloid cell proliferation. Instead, it exhibits a biphasic differentiation of basaloid epithelium composed of luminal ductal cells and abluminal cells arranged in a palisaded pattern and often surrounded by a thickened basement membrane. In addition, a subset of BCA is characterized by the abundant S100 protein–positive possibly “myoepithelial cell–derived stroma,” making this neoplasm in fact triphasic and distinctive. This tumor entity has been described for the first time already in 1986 by Dardick et al. [2]. Recently, Jurmeister et al. proposed a DNA methylation–based classifier of salivary gland tumors [13], demonstrating that myoepithelioma and pleomorphic adenoma form a uniform epigenetic class, supporting the theory of a single entity with a broad but continuous morphologic spectrum. However, specific epigenetic signatures of BCA were not addressed in their study [13].

BCA has been reported to harbor hotspot point mutations in the *CTNNB1* gene, which encodes β-catenin [16]. The mutation inhibits the degradation of β-catenin and activates the Wnt pathway [17]. The detection of nuclear β-catenin accumulation by immunohistochemical staining is another specific feature of BCA and has been included in the desirable diagnostic criteria in the 2024 WHO Tumour Classification [1]. Furthermore, because S100-positive stromal cells also show the nuclear expression of β-catenin, they are considered to be neoplastic cells [18].

The presence of gain-of-function *CTNNB1* gene alterations lying within ubiquitination recognition motif [19] was identified in 11 out of 17 cases, while 3 cases were either unanalyzable or not tested, and 3 cases (cases 7, 10, and 11) showed no *CTNNB1* gene mutation. However, nuclear expression of β-catenin was observed in these 3 cases. This phenomenon may be explained by different types of alteration in *CTNNB1* or other genes in the Wnt pathway that were not covered by the NGS panel used. Possible mechanisms include different activating alterations in upstream regulators of *CTNNB1* (or even directly *CTNNB1*) [20–22] as well as the disruption of negative regulators, including epigenetic alterations, of the Wnt pathway [22].

Despite widespread application of molecular testing to reclassify many existing salivary tumors and define new tumor types, the molecular underpinnings of BCA with abundant S100 protein–positive stroma have never been fully investigated. In this study, we performed next-generation sequencing and DNA methylation analysis on a cohort of BCA with abundant S100 protein–positive stroma and compared it with conventional BCA and cellular PA/ME to better understand their pathogenesis and possible relationships. The clustering algorithms identified two distinct tumor entities, partially overlapping with the pre-analytic phase of the investigation. The methylation data align with biologic features: CTNNB1-mutant BCAs segregate with S100-positive stromal BCAs, whereas BCAs lacking this mutation cluster with pleomorphic adenomas.

In conclusion, these findings provide molecular confirmation that a subset of BCA with S100 protein–positive stromal cells, as described earlier by Dardick, is a distinctive triphasic salivary gland entity, and it does not represent a variety of cellular PA or any type of hybrid lesion between BCA and PA. These findings suggest that BCAs are tumors that morphologically display tricellular differentiation into inner (luminal) ductal epithelial cells, outer (abluminal) basaloid myoepithelial cells, and spindle-shaped stromal S100-positive cells (stromal abluminal). However, this study did not address whether stromal S100-positive cells, as suggested by a previous ultrastructural study, are of myoepithelial origin. Further studies are needed to verify these findings.

## Data Availability

Data supporting the findings of this study are available within the article. The complete datasets generated during and/or analyzed during the current study are available from the corresponding author upon reasonable request.
